# Women with spondyloarthritis occurring in the peripartum period develop a singular phenotype

**DOI:** 10.1186/s12891-026-10070-1

**Published:** 2026-06-19

**Authors:** Baptiste de Maleprade, Baptiste Gérard, Pauline Brevet, Camille Prum-Delepine, Gilles Avenel, Nicolas Sens, Sophie Pouplin, Stéfan Jacques Darmoni, Julien Grosjean, Olivier Vittecoq, Thierry Lequerré

**Affiliations:** 1 Department of Rheumatology & CIC-CRB 1404, Univ Rouen Normandie, Normandie Univ, CHU Rouen, CHU de Rouen, 1 rue de Germont, 76031 Rouen cedex, Rouen, F-76000 France; 2 Regenerative Medicine and Skeleton Laboratory, RMeS, UMR 1229, Nantes Université, CHU Nantes, INSERM, Regenerative Medicine and Skeleton, Nantes, F-44000 France; 3 Department of digital health, CHU Rouen, LIMICS, INSERM U1142 Sorbonne Universités and Sorbonne Paris Nord, Rouen, France

**Keywords:** Spondyloarthritis, TNFα inhibitor, Parturition, Peripartum

## Abstract

**Background:**

Spondyloarthritis (SpA) has specific features in women and may be triggered by parturition. The peripartum period is marked by musculoskeletal remodelling and IL-17 secreting cell activation to enable childbirth. We therefore hypothesized that peripartum SpA might predominantly involve the entheses and be less dependent on HLA-B27 and TNFα.

**Methods:**

We conducted a retrospective, observational, case-control study using the Rouen University Hospital’s clinical data warehouse. Delivery dates and symptom onset were available for 154 women with SpA. Characteristics were compared between cases (with peripartum SpA) and controls.

**Results:**

We identified 58 cases and 96 controls. HLA-B27 (37.3% vs. 52.3%, p-value: 0.04), anterior chest syndrome (74.1% vs. 54.7%, p-value: 0.012 and psoriasis (37.9% vs. 16.8%, p-value: 0.004 were significantly associated with peripartum SpA. Lower rates of biological inflammatory syndrome at TNFα inhibitor introduction, (22.2% vs. 48.1%, p-value: 0.003), of radiographic sacroiliac damage (4% vs. 18.6%, p-value: 0.012 and of MRI sacroiliitis (30.6% vs. 47.4%, p-value: 0.046 were observed in women with peripartum SpA. Also, a lower retention rate of TNFα inhibitors was observed in women with peripartum SpA, 45.6% at 12 months, versus 71.2% controls (Odd ratio: 2.56 [95% CI: 1.34–4.95]). Multivariate survival analysis found a hazard ratio for TNFα inhibitor discontinuation of 2.43 (95% CI: 1.62–3.65) within the first two years of treatment.

**Conclusions:**

Our study highlights a distinct phenotype of peripartum SpA, and a lower retention rate of TNFα inhibitors suggesting a worse response to these class of bDMARD.

**Trial registration:**

As we conducted a retrospective study without health care intervention, no prospective registration was done.

## Introduction

Spondyloarthritis (SpA) is a rheumatic disease characterised by inflammatory involvement of the entheses [1]. The prevalence of SpA in Europe is 0.5% [2], with a balanced sex ratio for axial forms without radiographic involvement but sex differences (male to female *ratio* 2:1) in peripheral or axial forms with radiographic involvement [3].

The inflammatory process of SpA begins in the entheses, and is dependent on mechanical stress and immune activation [4, 5]. The IL-17-secreting cells most commonly found in peripheral joints are mast cells [6, 7], neutrophils [7] and Tγδ lymphocytes [8]. Macrophages and CD8 + T lymphocytes, TNFα-producing cells [9], have been identified in numerous human axial samples [10] as well as in peripheral samples [11].

In women, compared to men, different authors observed a more severe involvement of the entheses [12, 13], a weaker association with HLA-B27, a less elevated CRP level [14, 15], and a stronger association with inflammatory bowel disease (IBD) and psoriasis, but a weaker association with uveitis. Imaging modalities found less structural damage on X-rays [14, 15] and more sacroiliac joints oedema on MRI in patients without proper SpA [16]. Women also had a poorer response to anti-TNF drugs [13, 17].

In women, 18–28% of onset of SpA occurs in the postpartum period [18–20] but no recent studies have updated these rates. Disease activity increases in the postpartum period in 30–50% of SpA [21, 22] and appears to return to normal one year after delivery [23]. From the 32nd week of amenorrhoea [24], to enable childbirth, modifications will progressively occur in the female pelvis driven by sexual and non-sexual hormones such as relaxin [25, 26] associated with the secretion of metalloproteinases targeting type I (main constituent of tendons and ligaments [27]) and III collagens [28]. These modifications, responsible for microlesions in these tissues, also involve the entire musculoskeletal system. Parturition involves the activation of macrophages, neutrophils, mast cells [28] and Tγδ lymphocytes [29], which produce pro-inflammatory mediators such as IL17 [30]. TNFα is minimally expressed by decidual macrophages [31]. We therefore hypothesized that SpA occurring in the peripartum period might predominantly involve the entheses and be less dependent on HLA-B27 (as immune activation is already occurring) and TNFα.

To our knowledge, there is no cohort investigating the characteristics of women with SpA occurring in the peripartum or postpartum period. The aim of this case-control study was to investigate the characteristics of SpA occurring in women in the peripartum period and their response to treatment.

## Materials and methods

### Design

This single center, retrospective, observational and case-control study was conducted at Rouen University Hospital, France.

### Setting

Patients were identified using EDSaN, Rouen University Hospital’s clinical data warehouse, which aggregates different fragmented healthcare data in French language from clinical information systems. More precisely, the system is able to search into unstructured data (e.g. clinical notes) dealing with nuances of natural language (e.g. negations) to maximize precision and recall [32]. We searched the queries (in French) « spondyloarthritis - ankylosing spondylitis - spondylarthritis - spondylarthropathy - sacroiliitis - psoriatic arthritis - SpA » with « pregnancy - postpartum », in all documents associated with consultations and hospitalizations from January 2000 to January 2023, containing rheumatology and obstetrics data. Files where symptom onset period and parturition date were mentioned by physicians, and that met our inclusion and exclusion criteria were then manually selected (*n* = 154).

### Study inclusion criteria

Inclusion criteria were women over 18 years of age at the time of symptom onset, diagnosed with SpA by a rheumatologist experienced in inflammatory diseases at a tertiary care centre between January 2000 and January 2023. The diagnosis of SpA had to be validated at the last follow-up visit. Exclusion criteria were the association with another inflammatory rheumatism, women meeting the 2016 ACR fibromyalgia diagnostic criteria [33] at diagnosis, onset of symptoms after voluntary termination of pregnancy or during pregnancy before the 36th week of amenorrhoea, or unavailability of period of symptom onset and delivery. We excluded 282 patients for missing data about SpA onset or parturition, 113 for final diagnosis other that SpA or fibromyalgia criteria at diagnosis, 24 for diagnosis of SpA before the year 2000, 13 for other inflammatory disease associated with SpA and seven for onset of disease during pregnancy or after abortion.

### Patients’ characteristics

Socio-demographic, clinical and biological characteristics were collected. Demographic characteristics included age at onset of symptoms, and smoking status. Clinical characteristics were collected from standardized observations and medical files and included family history of SpA, clinical involvement of sacroiliac joints, of spine, history of arthritis, enthesitis (anterior chest wall pain, calcaneal enthesitis, enthesitis at any site; entheses were clinically checked), dactylitis, arthralgia, uveitis, IBD, psoriasis, non-steroidal anti-inflammatory drug (NSAID) intake. Biological characteristics included history of biological inflammation (defined as a C reactive protein (CRP) level of > 5 mg/L), CRP at diagnosis, CRP at introduction of TNFα inhibitors, and HLA-B27 status. In addition, imaging data were collected from clinical reports: structural damage on X-ray (which were carried out at the clinician’s discretion during follow-up), and sacroiliitis on MRI (evaluation at diagnosis). Sacroiliitis on MRI was defined following the ASAS recommendation [34], and equivocal cases were jointly reviewed by a rheumatologist and a radiologist. Treatment response was assessed according to the following parameters: retention rates of the TNFα inhibitors, etanercept, infliximab, adalimumab, golimumab, and certolizumab, and Bath Ankylosing Spondylitis (BASDAI) and Bath Ankylosing Spondylitis (BASFI) scores before and 6 months after the introduction of TNFα inhibitors. Treatments of less than one month were not analyzed, and data were censored after 24 months of follow-up. We determined the proportion of women meeting the following classification criteria: axial and peripheral ASAS classification criteria [35], New York criteria [36], Amor criteria [37] and ESSG criteria [38].

### Cases and controls

Cases were defined as women with onset of symptom in the period one month before delivery (in agreement with the onset of immunological and collagen remodelling mechanisms) and one year after delivery (in agreement with the date of return to basal activity of postpartum SpA flares) (*n* = 58). Women whose symptoms occurred outside this period were included as controls (*n* = 96), so they were either nulliparous women or develop symptoms 1 year after delivery. Treatment dosages followed the recommendations of the French regulatory authorities.

### Analysis

Statistical analyses and graphs were generated using Python (Python Software Foundation - packages: scikit learn 1.1, matplotlib 3.6.0, seaborn 0.12.1, lifelines 0.27.7, sksurv 0.21.0). Quantitative variables were compared using Mann-Whitney or t-test (depending on distribution and number of patients to analyze) and qualitative variables using Boschloo tests. Survival analyses at selected time points were performed using log-rank test, after a log(-log(x)) transformation to increase statistical power [39]. We performed the multivariate survival model using Cox regression on variables pre-selected from the literature (CRP at TNFα inhibitor introduction and sacroiliitis on MRI [40]). P-values below 0.05 were considered significant, except for the multivariate analysis where Bonferroni correction was applied and a threshold of 0.017 was used.

### Ethics approval

Our study was conducted in accordance with the Declaration of Helsinki and approved by the local ethics committee (CSE – EDSaN) for research on existing data at Rouen University Hospital (2021/0190/OB), approval need was waived by this comity. Moreover, the EDSaN data warehouse has been approved by the CNIL French agency (DT-2020-007, 19th october 2020).

## Results

### Comparison of clinical characteristics between women with spondyloarthritis in the peripartum period and controls

In total, 154 women were diagnosed with SpA in our centre between January 2000 and January 2023. Among them, 58 women with SpA occurring in the peripartum period were included as cases and the remaining 96 women were included as controls. Their demographic characteristics are summarised in Table 1. The mean age at disease onset was slightly but significantly higher in the peripartum group compared with the control group (28.7 ± 5.1 years versus 26.1 ± 6.8 years respectively). The delay to diagnosis was longer in the peripartum group compared to controls, 40.9 ± 50.4 months versus 28.5 ± 31.1 months, respectively. A lower rate of HLA-B27 was observed in the peripartum group compared to controls, 37.3% versus 52.3%, respectively (p-value: 0.04), with no difference in family history of SpA. A majority of women had axial pain, spinal pain, and peripheral arthralgia. There was a trend for more enthesitis in the peripartum group compared to controls, with a significant difference in anterior chest wall pain, 74.1% versus 54.7% respectively (p-value: 0.012). A higher rate of psoriasis was observed in the peripartum group compared to controls, 37.9% versus 16.8%, respectively (p-value: 0.004).

**Table 1:** Demographic, clinical and biological characteristics of the 154 women included

	Women with SpA in the Peripartum period (*n* = 58)	Controls (*n* = 96)	All patients (*n* = 154)	Missing data	*p*-value
Age, mean ± SD (years)	28.7 ± 5.1	26.1 ± 6.8	27.1 ± 6.3	3 (1.9%)	0.013
Smoker, n (%)	15 (44.1)	40 (58.8)	55 (53.9)	52 (33.8)	0.174
HLA-B27, n (%)	19 (37.3)	47 (52.3)	66 (42.9)	14 (9.1)	
Family history of SpA, n (%)	14 (29.2)	23 (29.1)	37 (29.1)	27 (17.5)	1.000
Nulliparous women at symptom onset	0 (0.0)	74 (77.1)	74 (48.1)	0 (0.0)	< 0.001
Any history of symptoms					
Buttock pain, n (%)	54 (93.1)	94 (97.9)	148 (96.1)	0	0.222
Spinal pain, n (%)	57 (98.3)	89 (92.7)	146 (94.8)	0	0.195
Arthralgia, n (%)	47 (81.0)	74 (78.9)	122 (79.7)	1 (0.6)	0.796
Peripheral arthritis, n (%)	8 (13.8)	14 (14.7)	22 (14.4)	1 (0.6)	0.941
Enthesopathy					
All sites, n (%)	53 (91.4)	78 (82.1)	131 (85.6)	1 (0.6)	0.086
Heel, n (%)	37 (63.8)	52 (54.7)	89 (58.2)	1 (0.6)	0.176
Anterior chest pain, n (%)	43 (74.1)	52 (54.7)	95 (62.1)	1 (0.6)	0.012
Dactylitis, n (%)	4 (6.9)	10 (10.5)	14 (9.2)	1 (0.6)	0.544
Anterior uveitis, n (%)	2 (3.4)	9 (9.5)	11 (7.2)	1 (0.6)	0.213
IBD, n (%)	4 (6.9)	15 (15.8)	19 (12.4)	1 (0.6)	0.124
Psoriasis, n (%)	22 (37.9)	16 (16.8)	38 (24.8)	1 (0.6)	0.004
Any history of biological inflammatory syndrome, n (%)	23 (41.1)	52 (54.7)	75 (49.7)	3 (1.9)	0.053
CRP at diagnosis, *n* > 5 (%)	23 (40.4)	39 (43.8)	62 (42.8)	9 (5.8)	0.666
CRP at diagnosis, mean ± SD (mg/l, among CRP > 5)	30.6 ± 43.6	39.7 ± 52.2	36.4 ± 49.0	9 (5.8%)	0.145

*CRP* C-reactive protein, *IBD* Chronic inflammatory bowel disease, *n* number, *SD* Standard deviation, *SpA* Spondyloarthritis

A lower rate of imaging joint damage was observed in the peripartum group compared to controls (Table 2), with structural damage on X-ray (at any sites, X-ray were carried out at the clinician’s discretion) found in 7.8% versus 20.3%, (p-value: 0.047), radiographic damage (sacroiliac joints) in 4% versus 18.6% (p-value: 0.012) and MRI sacroiliitis in 30.6% versus 47.4% (p-value: 0.046).

**Table 2:** Radiographic and MRI involvement of women with SpA in the peripartum period and controls

	Peripartum SpA (*n* = 58)	Controls (*n* = 96)	*p*-value
Radiographic damage at any site, *n* (%)	4 (7.8)	15 (20.3)	0.047
Radiographic damage of sacroiliac joint, n (%)	2 (4.0)	13 (18.6)	0.012
Sacro-iliitis on MRI at diagnosis, n (%)	15 (30.6)	36 (47.4)	0.046

*n* number, *SpA* Spondyloarthritis

### Classification criteria for women with SpA in the peripartum period and controls

The diagnosis of SpA had to be confirmed at the last follow-up visit. The mean duration of follow-up for all groups was 85.0 ± 64.7 months. Among the classification criteria (Table 3), 100% of our population met the ESSG criteria. Consistent with the findings on HLA-B27 and MRI sacroiliitis, only 38% of women in the peripartum group met the axial ASAS criteria, versus 65.6% of controls (p-value: 0.003). Similarly, the New York criteria were met by 3.8% versus 17.9% (p-value: 0.02) and 83.3% versus 99.0% for the Amor criteria (p-value < 0.001). Peripheral ASAS criteria were met in equal proportions in the peripartum group and controls,73.5% versus 71.6%. We found that 98.1% of women in the peripartum group met at least two sets of criteria among ESSG, ASAS, Amor and New York versus 99% of controls. Only one woman in each group met only one set of criteria.

**Table 3:** Classification criteria of women with SpA in the peripartum period and controls

	SpA in the Peripartum period (*n* = 58)	Controls (*n* = 96)	*p*-value
	Follow-up, mean ± SD (months)	77.3 (59.5)	89.6 (67.6)
Axial ASAS criteria, n (%)	19 (38.0)	61 (65.6)	0.003
Peripheral ASAS criteria, n (%)	36 (73.5)	68 (71.6)	0.847
New York criteria, n (%)	2 (3.8)	12 (17.9)	0.021
Amor criteria, n (%)	45 (83.3)	95 (99.0)	< 0.001
ESSG criteria, n (%)	56 (100.0)	96 (100.0)	1.000
At least 2 criteria, n (%)	51 (98.1)	95 (99.0)	

*ASAS* Assessment of SpondyloArthritis international Society, *ESSG* European spondylarthropathy study group, *n* number, *SpA* Spondyloarthritis

### Treatment response of women with SpA in the peripartum period and controls

**Fig. 1:**
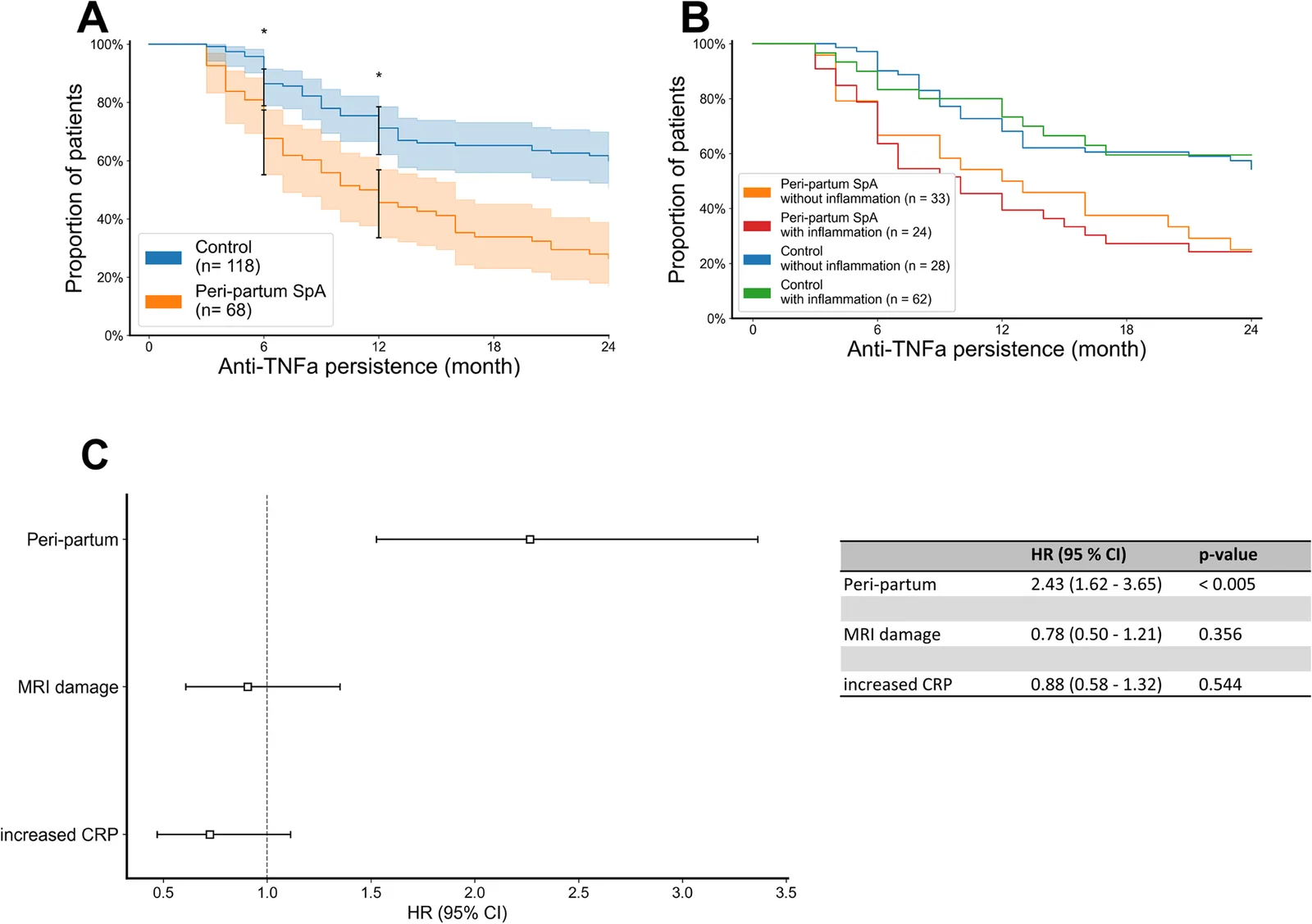
TNFα inhibitor retention rate for women with SpA in the peripartum period and controls. Comparison between women with SpA in the peripartum period and controls (**A**). Stratified representation on inflammatory sign (sacroiliitis on MRI and/or increased CRP before TNFα inhibitor introduction) and symptom onset period (**B**). Multivariate analysis of TNFα inhibitor retention rate based on sacroiliitis on MRI, increased CRP before TNFα inhibitor introduction and symptom onset period (**C**). CI: confidence interval; HR: hazard ratio; Spa: spondyloarthritis; *: p-value < 0.05

We identified 68 TNFα inhibitors in 32 women in the peripartum group and 123 TNFα inhibitors in 70 controls (Table 4). CRP level before TNFα inhibitor introduction, a known predictor of therapeutic response, was increased in 22.2% of women with peripartum SpA versus 48.1% of controls (p-value: 0.003). The delay between the onset of symptoms and TNFα inhibitor introduction was shorter in the peripartum group compared to the control group. Kaplan-Meier assessment of the retention rate of TNFα inhibitors at 12 months (Fig. 1A) shows a significant difference between groups: 45.6% (95% CI: 33.5–56.9) of women with peripartum SpA retained their treatment, compared with 71.2% (95% CI: 62.1–78.5) of controls (p-value < 0.001). This difference in treatment retention rate was already significant at 6 months, 67.6% (CI 95%: 55.1–77.4) in the peripartum group and 86.4% (CI 95%: 78.8–91.5) in the control group (p-value < 0.001). At 24 months, the retention rate of TNFα inhibitors was 26.5% (CI 95%: 16.7–37.3) in the peripartum group and 59.9% in controls (CI 95%: 50.5–68.2). The odds ratio for retaining TNFα inhibitors at 12 months according to the onset of symptoms in the peripartum period or not was 2.56 (CI 95%: 1.34–4.95).

**Table 4:** Therapeutic evaluation of women with SpA in the peripartum period and controls

	Women with SpA in the peripartum period (*n* = 58)	Controls (*n* = 96)	*p*-value
CRP before biotherapy, *n* > 5 (%)	10 (22.2)	38 (48.1)	0.003
CRP before biotherapy, mean ± SD (mg/l among *n* > 5)	16.1 ± 43.6	27.7 ± 34.6	0.333
NSAID intake, n (%)	42 (72.4)	69 (75.0)	0.713
TNFα inhibitor therapy (n patients)	68 (32)	123 (70)	
BASDAI before treatment, mean ± SD	6.74 ± 1.41	5.55 ± 1.53	
BASFI before treatment, mean ± SD	5.30 ± 2.00	4.66 ± 2.14	
Time to initiation of bDMARD in months, mean (SD)	63.3 ± 47.4	84.1 ± 63.3	0.033
6-months follow-up after biotherapy initiation			
BASDAI50 responder, n (%)	0 (0.0)	11 (22.4)	
Low BASDAI activity, n (%)	7 (13.7)	28 (47.4)	
BASDAI improvement, mean ± SD	− 0.64 ± 2.04	-1.13 ± 1.90	
BASFI improvement, mean ± SD	− 0.40 ± 2.07	-1.21 ± 2.25	
Treatment retention rate at 12 months, n (%)	31 (45.6)	84 (68.3)	0.002

No statistical tests were carried out on the BASDAI and BASFI data due to the high number of missing data.

*n* number, *SD* Standard deviation, *SpA* Spondyloarthritis

Before TNFα inhibitor introduction, BASDAI and BASFI scores were higher: 6.74 (standard deviation: ±1.41) in the peripartum group compared to controls, 6.74 ± 1.41 versus 5.55 ± 1.53, respectively. At 6-month follow-up, a worse treatment response was observed in women in the peripartum group with no evaluable patient reaching BASDAI50 (50% decrease in BASDAI) (Table 4) and only 13.7% reaching a low level of disease activity (defined as a BASDAI score of less than 4), compared with 47.4% of controls for whom data were available. The mean decreases in BASDAI and BASFI scores were respectively − 0.64 ± 2.04 and − 0.40 ± 2.07 in the peripartum group, and − 1.13 ± 1.90 and − 1.21 ± 2.25 in the control group. A stratified representation of retention rate on the presence of at least one objective inflammatory parameter (CRP greater than 5 mg/L prior to TNFα inhibitor introduction and/or sacroiliitis on MRI) shows an overlay of the curves for the inflammatory and non-inflammatory groups (Fig. 1B).

Finally, we performed a multivariate analysis of TNFα inhibitor discontinuation over 24 months using a Cox model. The variables analysed were a CRP level greater than 5 mg/L before TNFα inhibitor introduction or sacroiliitis on MRI, and peripartum SpA status (Fig. 1C). The hazard ratio (HR) was 0.78 (95% CI: 0.50–1.21) for MRI (p-value: 0.356) and 0.88 (95% CI: 0.58–1.32) for CRP (p-value: 0.544). Although not significant, there was a trend in our cohort for these two variables to favour retention rate. The HR for peripartum SpA status was 2.43 (95% CI: 1.62–3.65, p-value < 0.005).

## Discussion

To our knowledge, this study is the first to describe the clinical, biological, and imaging features and the therapeutic response of women with SpA occurring in the peripartum period. We were able to identify in this population a weaker association with HLA-B27, more enthesitis and notably more anterior chest wall pain, less radiographic joint involvement and sacroiliitis on MRI compared with other women with SpA. These features meet the specificities of female SpA already described [12–15], which in our study seemed to predominate in the peripartum SpA group. The prevalence of HLA-B27 (72.2% in women from the DESIR cohort [15] - “*devenir des spondylarthropathies indifférenciées récentes*”, a French national SpA cohort) was lower in women with peripartum SpA than in controls (37.3% and 52.3% respectively). Unlike our cohort, the DESIR cohort only analyzed SpA with axial involvement. But since more than 98% of our patients had inflammatory back pain, comparison was reasonable. In contrast, anterior chest wall pain was involved in 44.6% of patients in the DESIR cohort [41] and in 54.7% of our controls. Its high prevalence in the peripartum SpA group (74.1%) might be explained by the histological structure of the manubrio-sternal joint. Indeed, this joint is the main site of anterior chest wall pain on MRI [42]. Together with the first rib, it forms a symphysis [43], composed of fibrocartilage rich in type I and III collagen [44] which are the main targets of metalloproteases responsible for cervical remodelling [24]. Serum levels of these metalloproteases in SpA are correlated with disease activity [45].

The association with psoriasis (17.1% in the DESIR cohort [41]), 16.8% in our controls, was markedly increased in the peripartum group (37.9%). Psoriasis is a highly IL-17-dependent skin disease, as demonstrated by the high efficacy of anti-IL17 in this condition [46]. In contrast, anti-IL17s have no proven efficacy in IBD or uveitis [47]. As mentioned above, IL17 may be one of the key factors driving the onset of disease in the peripartum period. We were unable to study the efficacy of anti-IL17 in our cohort and this will need further investigation.

The low prevalence of objective signs of inflammation, such as a sustained increase in CRP, or the absence of damage visible on imaging, may challenge the accuracy of the initial diagnosis. As pregnancy and parturition have a major physical and hormonal impact of pregnancy, it can trigger pain of various etiologies. Pregnancy-related pelvic pain, the origins of which are a complex combination of hormonal, biomechanical, metabolic and genetic actions, are grouped under the terminology “pregnancy-related pelvic girdle pain” [48] and highlights the significant changes in the patients’ musculoskeletal structures. The modification of the MRI signal of sacroiliacs joints from postpartum women, that can last up to two years, provides a good demonstration of these changes [49]. Traumatic pain, such as damage to the coccyx or ruptured symphysis [48], can also occur in the postpartum period and last for several months. However, careful clinical and iconographic analysis can help establish the diagnosis. Around 5% of such pain persists after childbirth [50]. These mechanical impairments may also contribute to the onset of SpA [5]. Fibromyalgia is frequently associated with SpA, especially in women, and may mimic its symptoms. Pregnancy and the postpartum have been described as exacerbating fibromyalgia [51], but we did not find any studies exploring its incidence in this period. The longer diagnosis delay in the peripartum SpA group could reflect the challenges in distinguishing these different diagnoses.

In our cohort, the biological inflammatory syndrome at diagnosis, the mean follow-up of 77.3 months and the positivity of at least two classification criteria in 98% of women with peripartum SpA (including ESSG, ASAS, New York and Amor criteria) are in favour of SpA. The association between the retention rate and the discontinuation of TNFα inhibitors and peripartum SpA status in the multivariate model (integrating sacroiliac MRI and CRP) is also a strong argument in favour of the importance of the latter. The low fulfillment of the ASAS criteria in the peripartum group could suggest that this set of classification is not appropriate for those SpA. As associations between peripheral enthesitis and anterior chest pain, the absence of HLA-B27, increased psoriasis and high BASDAI and BASFI scores were previously described, the phenotype of peripartum SpA seems to fit the same pattern [52]. The lack of difference in biological inflammatory syndrome at diagnosis, followed by a decrease in inflammation before TNFα inhibitor introduction in the peripartum group compared with controls may suggest an initial activation of the immune system, followed by a chronic sequellar phase. The activation of the immune system predominant in the initial phase of the disease could be another explanation for the lower retention rate of TNFα inhibitors.

The occurrence of SpA in the peripartum period was associated with a lower retention rate of TNFα inhibitors. At 12 months, the retention rate of women with peripartum SpA was 45.6% (Odds ratio: 2.56 [CI 95%: 1.34–4.95]) suggesting a low therapeutic response. Multivariate analysis using Cox models confirmed this negative association, finding a hazard ratio of 2.43 for the probability of TNFα inhibitor discontinuation in the first two years of treatment. Breast-feeding during the peripartum period may lead women to stop taking anti-TNFa. However, given the long time to diagnosis and introduction of biotherapy in our cohort, this was of only marginal importance. Sacroiliitis on MRI and elevated CRP, widely validated features of TNFα inhibitor success, could have their impact lowered in our female cohort where they are less common. On the contrary, as our cohort focused on peripartum SpA, the importance of this feature could be lowered in real practice depending on peripartum SpA incidence. In psoriatic arthritis, female sex was associated with a lower retention rate of TNFα inhibitors [17]. The retention rate after 2 years was 49.7% in men and 37.1% in women in the Danish Health Care Registers. In our study, the retention rate was 59.9% in controls and only 26.5% in women with SpA in the peripartum period after 2 years, which might suggest that removing the peripartum period could allow them to recover a maintenance level comparable with that of men. As treatments of less than one month were not analyzed, allergic reaction to TNFα inhibitor were not considered, and no adverse event occurred in our cohort: TNFα inhibitor discontinuation was due to inefficacy. This low retention rate could be due to a different pathophysiology in peripartum SpA, such as higher mechanical stress in enthesitis or IL-17 implication.

Our study also has limitations. As with any retrospective study, there can be much selection bias. The frequency of peripartum SpA cannot be assessed here. Moreover, because the onset of symptoms was often reported as a time period rather than a precise date, we were unable to analyze the interval between symptom onset and parturition. Our method of query using keywords probably inflated number of peripartum SpA compared to control. In a disease with potential intricated fibromyalgia symptoms, therapeutics response can be tricky to evaluate. Nevertheless, we made sure to exclude obvious diagnosis of fibromyalgia associated at diagnosis. The missing data (especially the WPI and SSS score) led us to use the ACR 2016 criteria for fibromyalgia, whose negativity was mainly determined by the following criteria in our cohort: “Generalized pain, defined as pain in at least 4 of 5 regions, must be present” and “Symptoms have been generally present for at least 3 months”. As a tertiary center specialized in auto immune disease, we decided to base SpA diagnosis on local clinician assumption. This decision made it possible to explore a wider range of clinical features, but could also increase the number of misdiagnosed patients. The amount of missing data also made it impossible to conclude on some results, such as treatment response assessed by BASDAI and BASFI scores. This response was nevertheless very low in women with peripartum SpA, as was the treatment retention rate.

Our work suggests that there is a distinct SpA phenotype in the peripartum period, with specific clinical features (more previous thoracic syndrome, more psoriasis), biological features (less biological inflammatory syndrome remote from diagnosis, a weaker association with HLA-B27), iconographic features (less radiographic and MRI damage) and therapeutic features (a lower retention rate of TNFα inhibitors). Peripartum onset could also be used as a negative predictor of treatment response. A better characterisation of SpA occurring in the peripartum period and of therapeutic response could lead to the development of specific treatments and contribute to the prevention of therapeutic wandering.

## Data Availability

The data underlying this article will be shared on reasonable request to the corresponding author.

## Abbreviations

BASFI: Bath Ankylosing Spondylitis
BASDAI: Bath Ankylosing Spondylitis
CI: confidence interval
CRP: C reactive protein
HR: hazard ratio
IBD: inflammatory bowel disease
N: number
NSAID: non-steroidal anti-inflammatory drug
SD: standard deviation
SpA: Spondyloarthritis
